# Supplementary material for: Systems genomics of salinity stress response in rice
Source: bioRxiv. 2024 Dec 22:2024.05.31.596807. Originally published 2024 Jun 3. Preprint. [Version 2] doi: 10.1101/2024.05.31.596807 (PMC11185513; doi:10.1101/2024.05.31.596807)
Supplement: 1 [file NIHPP2024.05.31.596807V2-supplement-1.pdf]

bioRxiv preprint doi: https://doi.org/10.1101/2024.05.31.596807; this version posted December 22, 2024. The copyright holder for this preprint (which was not certified by peer review) is the author/funder, who has granted bioRxiv a license to display the preprint in perpetuity. It is made available under aCC-BY 4.0 International license.

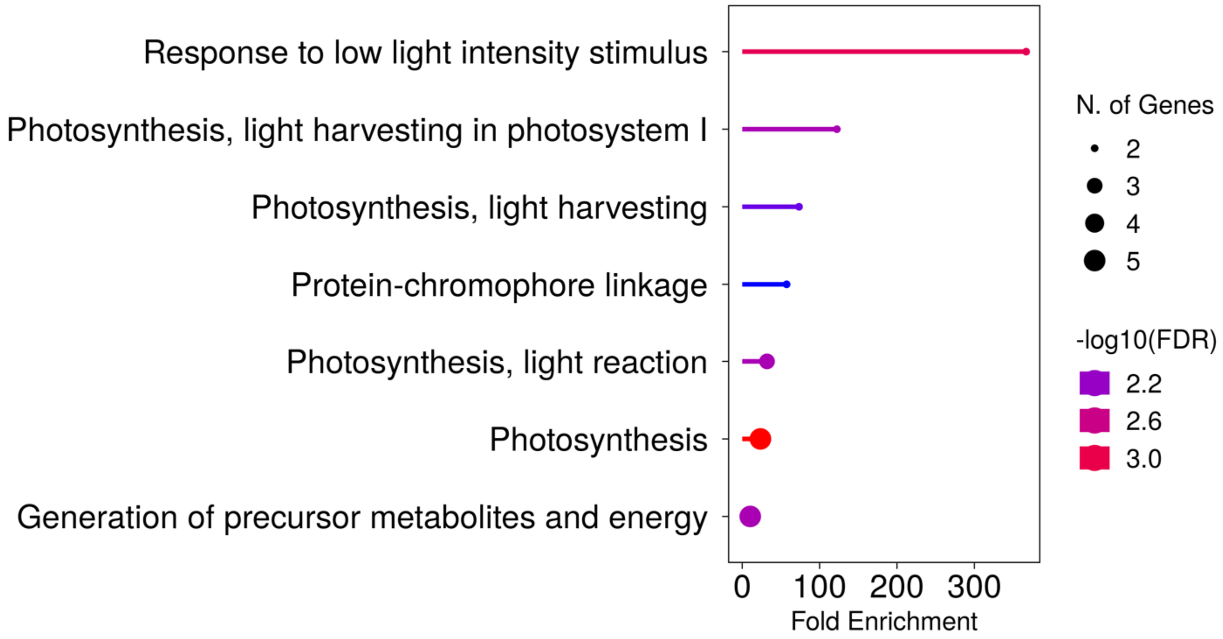


**Figure S1**: Pathway enrichment of the 51 antagonistically pleiotropic genes beneficial in normal conditions but detrimental in salinity stress conditions. Enrichment statistics provided in Supplementary Table 4.

bioRxiv preprint doi: https://doi.org/10.1101/2024.05.31.596807; this version posted December 22, 2024. The copyright holder for this preprint (which was not certified by peer review) is the author/funder, who has granted bioRxiv a license to display the preprint in perpetuity. It is made available under aCC-BY 4.0 International license.

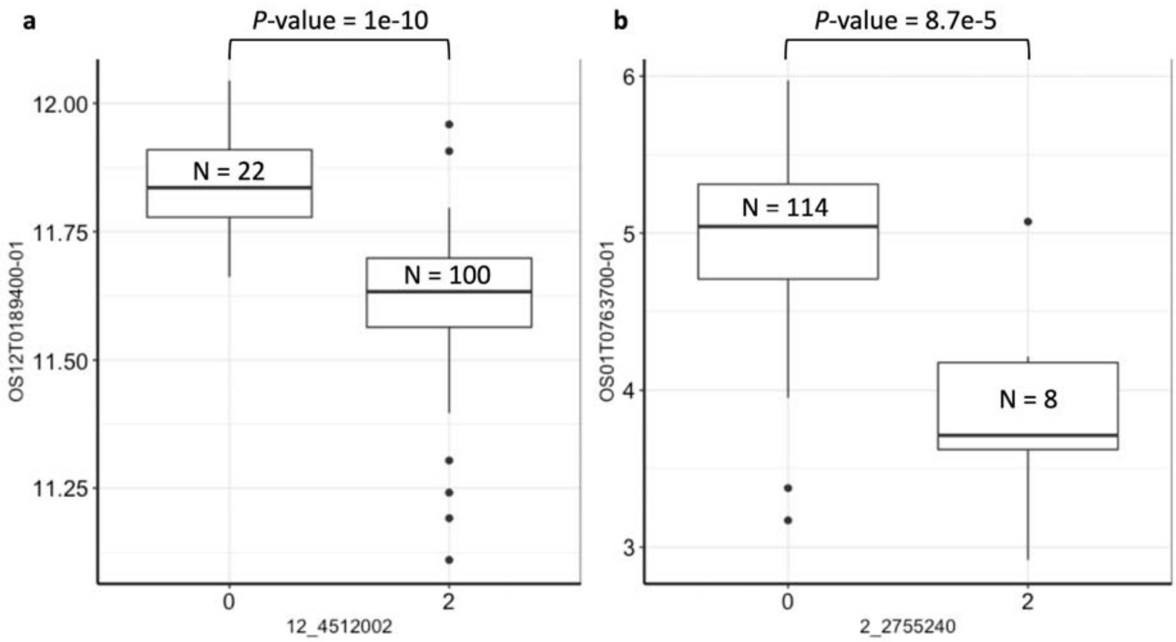


**Figure S2**: Boxplot representation of eQTLs for the two photosynthesis-related AP (antagonistically pleiotropic) genes beneficial in normal conditions. a, *cis*-eQTL (SNP = Chr12:4512002) for *PSAN* (OS12T0189400-01). b, *trans*-eQTL (SNP = Chr02:2755240) for *CRR7* (OS01T0763700-01). eQTL statistics provided in Supplementary Table 10. X-axis represents the eQTL SNP haplotypes, y-axis represents normalized transcript expression in normal conditions. Numbers inside box plots represent the number of accessions in each group. *P*-value indicates the one-sided Wilcoxon-test significance.

bioRxiv preprint doi: https://doi.org/10.1101/2024.05.31.596807; this version posted December 22, 2024. The copyright holder for this preprint (which was not certified by peer review) is the author/funder, who has granted bioRxiv a license to display the preprint in perpetuity. It is made available under aCC-BY 4.0 International license.

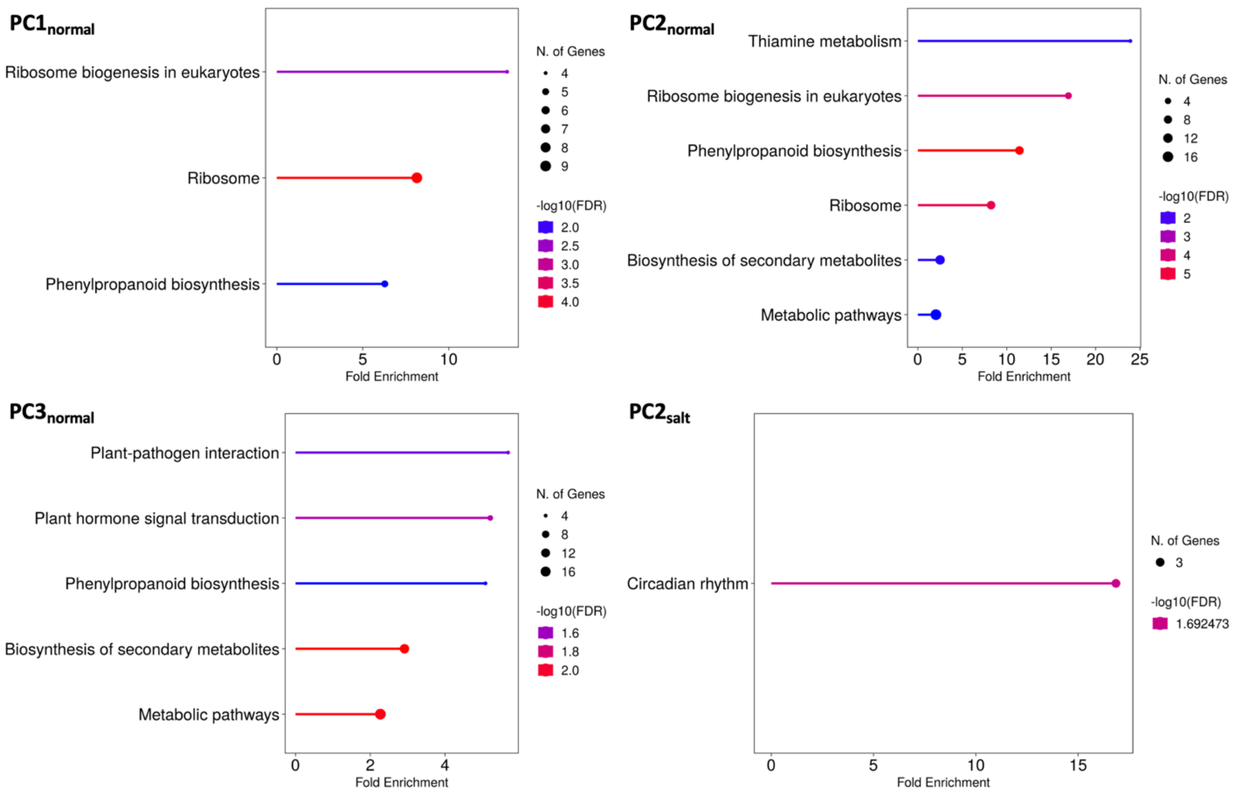


**Figure S3**: Enrichment of the suite of transcripts (*1% tails of the distributions of transcripts' loading values on principal components*) with significant selection gradients in both normal and salinity stress conditions.

bioRxiv preprint doi: https://doi.org/10.1101/2024.05.31.596807; this version posted December 22, 2024. The copyright holder for this preprint (which was not certified by peer review) is the author/funder, who has granted bioRxiv a license to display the preprint in perpetuity. It is made available under aCC-BY 4.0 International license.

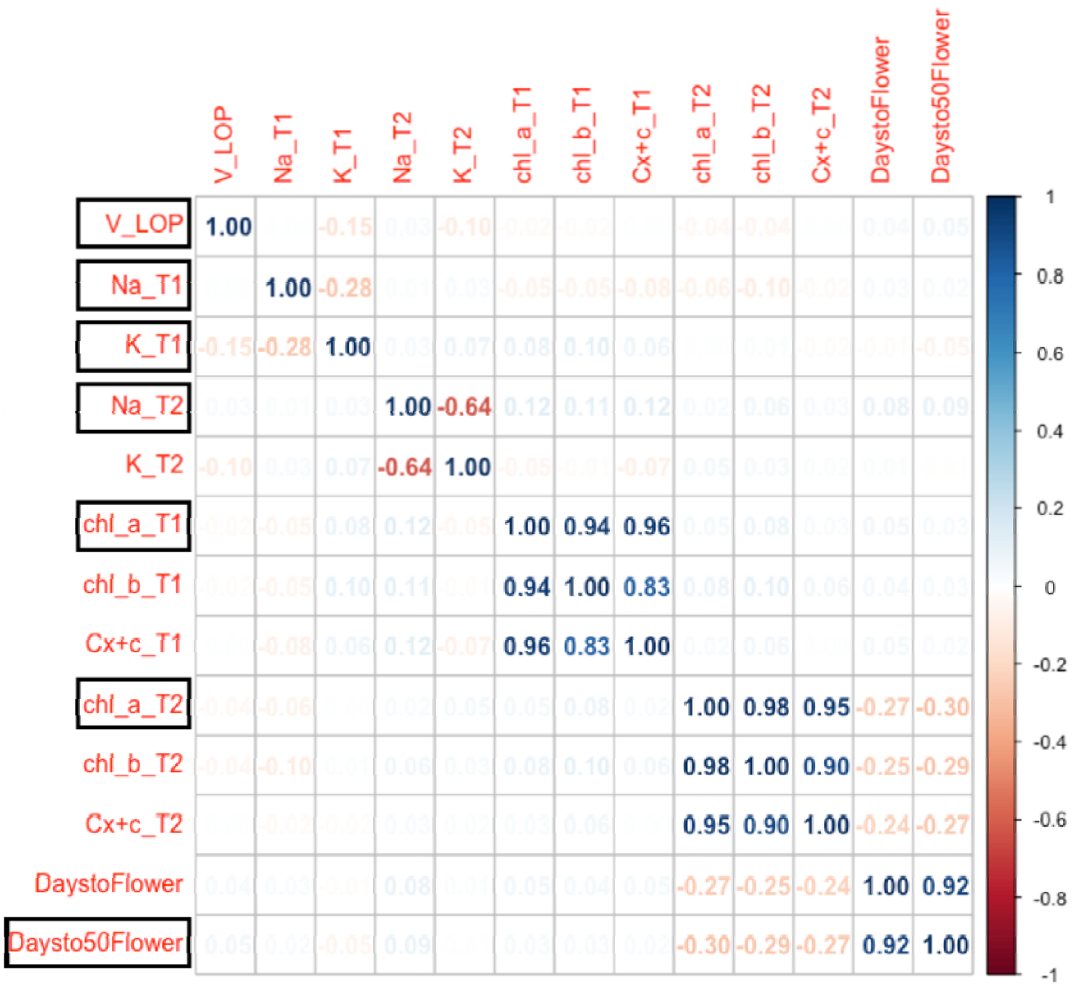


**Figure S4:** Correlation among functional traits in normal conditions. Highlighted black boxes indicate the uncorrelated traits chosen for selection analyses. Numbers inside the boxes and the heatmap represent Pearson correlation coefficients.

V_LOP: Leaf Osmotic Potential; Na: Sodium content; K: Potassium content; chl_a and chl_b: Chlorophyll a and b content, respectively; Cx+c: Total carotenoid content; DaystoFlower: First day of flowering; Daysto50Flower: Day on which 50% of plants in a plot flowered (focal plant and its nine neighboring plants); T1 and T2 represent vegetative and reproductive timepoints, respectively.

bioRxiv preprint doi: https://doi.org/10.1101/2024.05.31.596807; this version posted December 22, 2024. The copyright holder for this preprint (which was not certified by peer review) is the author/funder, who has granted bioRxiv a license to display the preprint in perpetuity. It is made available under aCC-BY 4.0 International license.

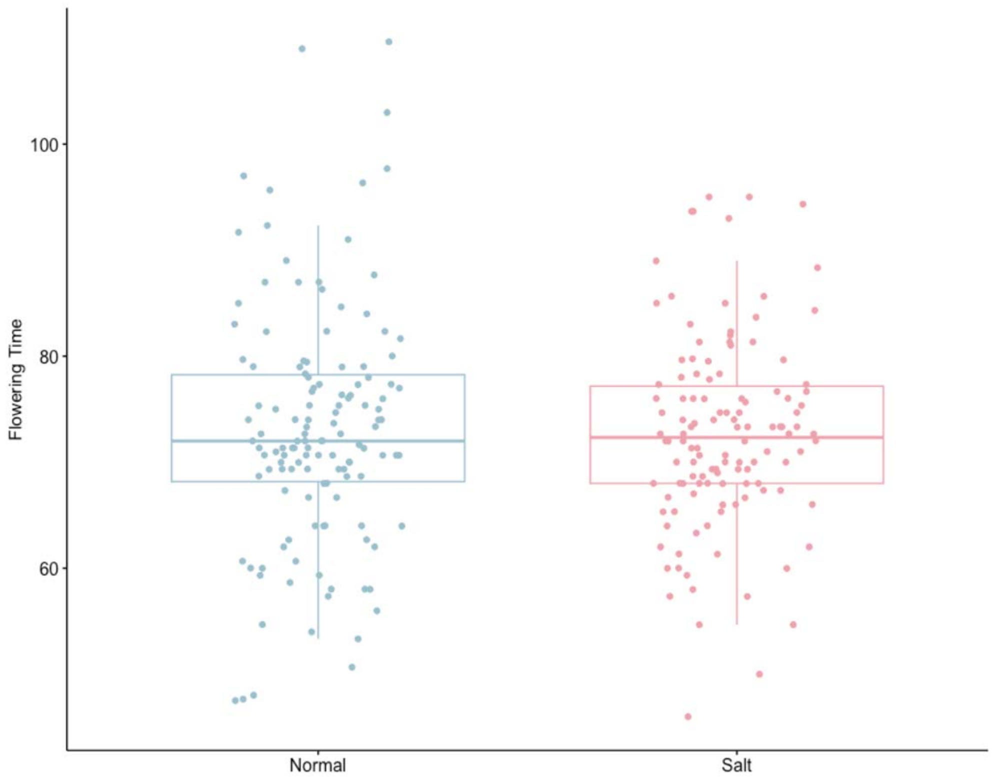


**Figure S5:** Distribution of flowering time (representing days to when 50% of plants in a plot flowered) in normal and salinity stress conditions. Paired t-test (two-sided paired t-test $P$ = 0.001) showed significant reduction in flowering times after salt treatment, indicating earlier flowering saline conditions.

bioRxiv preprint doi: https://doi.org/10.1101/2024.05.31.596807; this version posted December 22, 2024. The copyright holder for this preprint (which was not certified by peer review) is the author/funder, who has granted bioRxiv a license to display the preprint in perpetuity. It is made available under aCC-BY 4.0 International license.

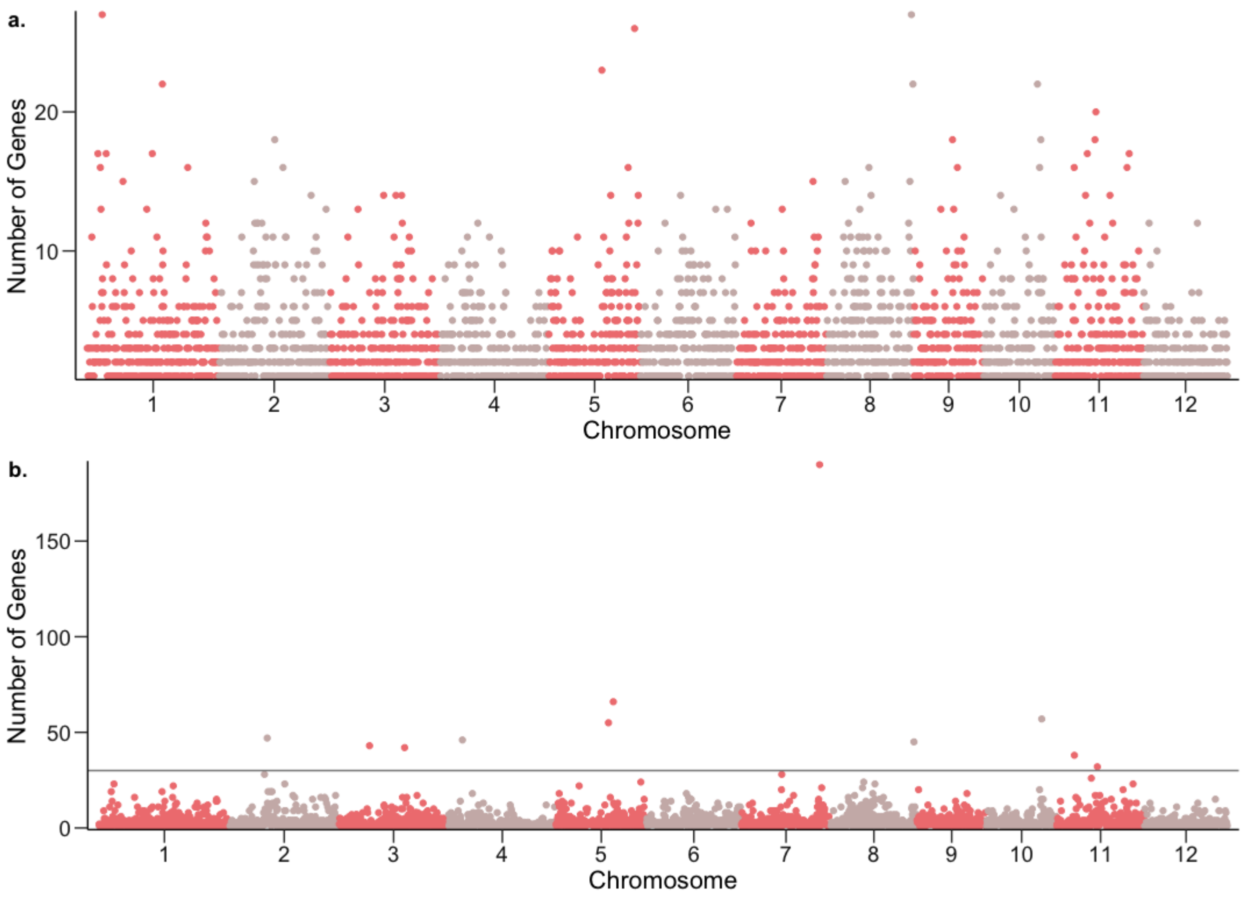


**Figure S6:** *Trans*-eQTL hotspots in normal (a.) and salinity stress (b.) conditions. X-axes indicate genomic locations of 100-kb nonoverlapping windows for which the total numbers of unique genes regulated were calculated (Y-axis). Hotspots were defined as windows regulating expression of over 30 genes; there were 0 hotspots in normal conditions (max number of genes = 28), and 11 hotspots in saline conditions. Gray horizontal line represents the cutoff of 30 genes.

bioRxiv preprint doi: https://doi.org/10.1101/2024.05.31.596807; this version posted December 22, 2024. The copyright holder for this preprint (which was not certified by peer review) is the author/funder, who has granted bioRxiv a license to display the preprint in perpetuity. It is made available under aCC-BY 4.0 International license.

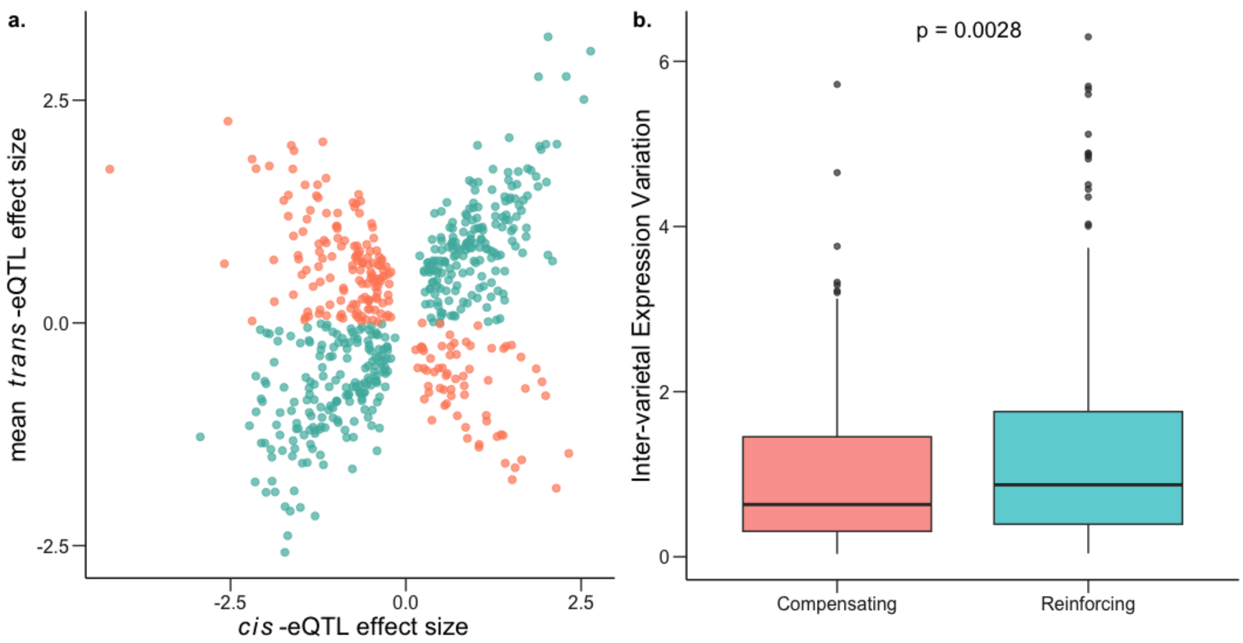


**Figure S7:** Compensating and reinforcing *cis-trans* effects in normal conditions**.** a, Effect sizes of genes with both *cis* and *trans* factors showing excess of *cis-trans* reinforcement (teal) in comparison to *cis-trans* compensation (salmon). b, Inter-varietal differences in gene expression for genes under compensating control is significantly lower than that for genes under reinforcing control; one-sided Mann-Whitney $P = 0.026$.

bioRxiv preprint doi: https://doi.org/10.1101/2024.05.31.596807; this version posted December 22, 2024. The copyright holder for this preprint (which was not certified by peer review) is the author/funder, who has granted bioRxiv a license to display the preprint in perpetuity. It is made available under aCC-BY 4.0 International license.

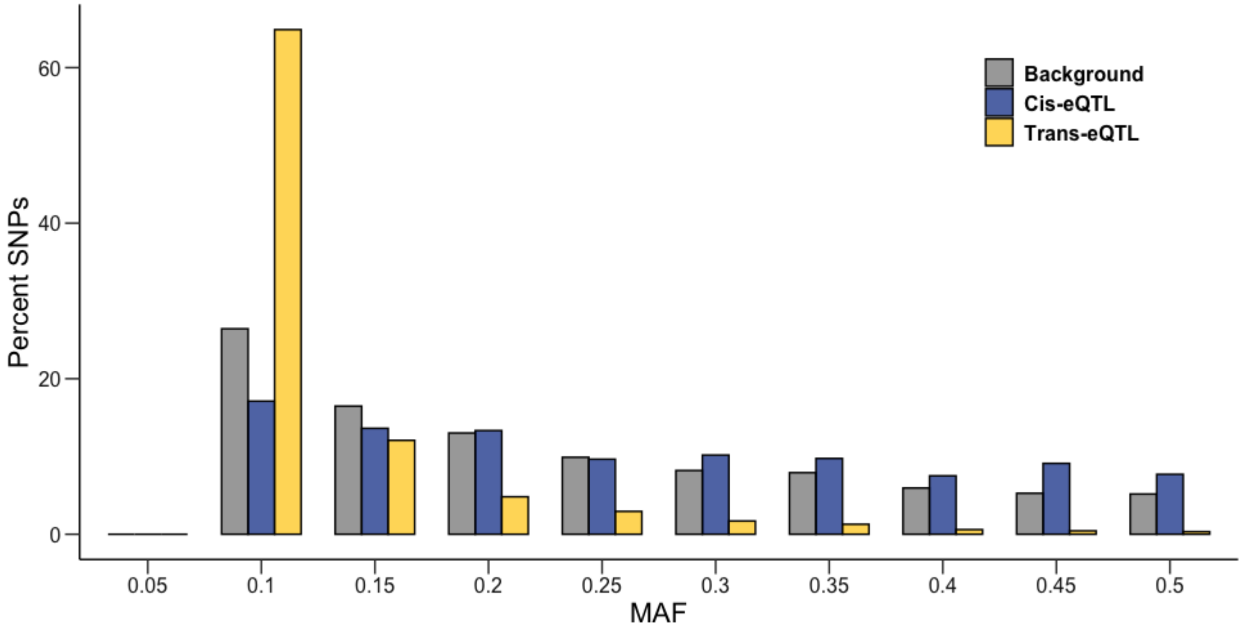


**Figure S8**: Frequency distribution of MAF (Minor Allele Frequency) for *cis*-eQTLs (blue) and *trans*-eQTLs (yellow) in normal conditions against the genome-wide background (gray).

bioRxiv preprint doi: https://doi.org/10.1101/2024.05.31.596807; this version posted December 22, 2024. The copyright holder for this preprint (which was not certified by peer review) is the author/funder, who has granted bioRxiv a license to display the preprint in perpetuity. It is made available under aCC-BY 4.0 International license.

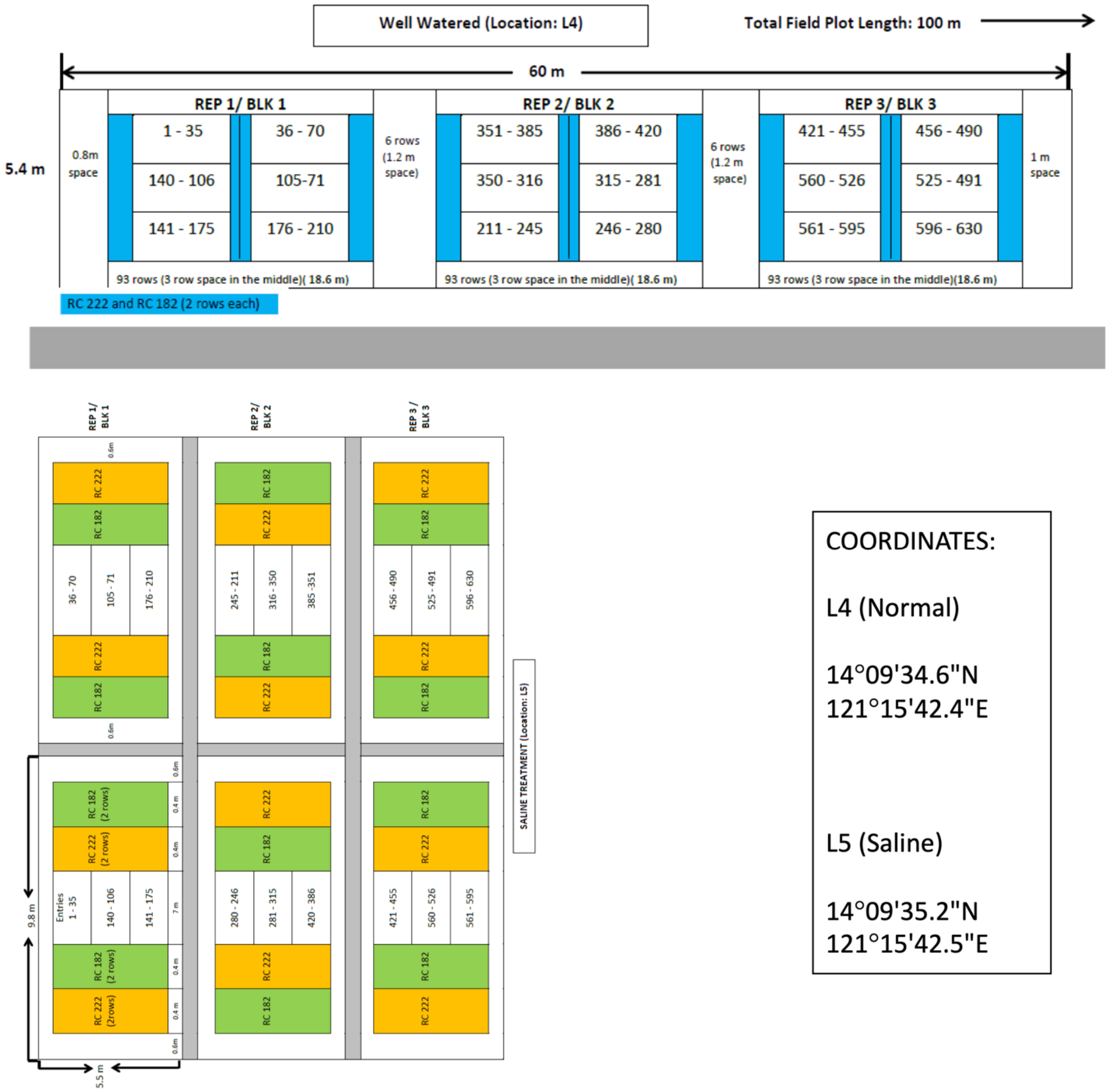


Figure S9: Experimental design for the field experiment. A total of 130 *O. sativa* ssp. *indica* and 65 *O. sativa* ssp. *japonica* accessions were selected for this experiment, including traditional varieties/landraces and three additional test varieties (Supplementary Table S12). Within each field environment (normal and saline), there were a total of three blocks each corresponding to a replicate, with each accession planted once per block, at a random location. Each experimental plot included the accessions NSIC Rc 222 and NSIC Rc 182 that served as border rows (Supplemental Table S13). REP: replicate; BLK: block.
